# Cellular Phone Use and Risk of Tumors: Systematic Review and Meta-Analysis

**DOI:** 10.3390/ijerph17218079

**Published:** 2020-11-02

**Authors:** Yoon-Jung Choi, Joel M. Moskowitz, Seung-Kwon Myung, Yi-Ryoung Lee, Yun-Chul Hong

**Affiliations:** 1 Department of Family Medicine and Center for Cancer Prevention and Detection, Hospital, National Cancer Center, Goyang 10408, Korea; hierica8@snu.ac.kr; 2 Department of Preventive Medicine, College of Medicine, Seoul National University, Seoul 110-744, Korea; 3 Environmental Health Center, College of Medicine, Seoul National University, Seoul 03080, Korea; 4 School of Public Health, University of California, Berkeley, Berkeley, CA 94720-7358, USA; jmm@berkeley.edu; 5 Department of Cancer Biomedical Science, National Cancer Center Graduate School of Cancer Science and Policy, Goyang 10408, Korea; 6 Division of Cancer Epidemiology and Management, National Cancer Center Research Institute, Goyang 10408, Korea; 7 Department of Occupational and Environmental Medicine, Seoul St. Mary’s Hospital of the Catholic University of Korea, Seoul 06591, Korea; hangingaround220@gmail.com; 8 Institute of Environmental Medicine, Seoul National University Medical Research Center, Seoul 03080, Korea

**Keywords:** cellular phone, electromagnetic field, tumor, case-control study, meta-analysis

## Abstract

We investigated whether cellular phone use was associated with increased risk of tumors using a meta-analysis of case-control studies. PubMed and EMBASE were searched from inception to July 2018. The primary outcome was the risk of tumors by cellular phone use, which was measured by pooling each odds ratio (OR) and its 95% confidence interval (CI). In a meta-analysis of 46 case-control studies, compared with never or rarely having used a cellular phone, regular use was not associated with tumor risk in the random-effects meta-analysis. However, in the subgroup meta-analysis by research group, there was a statistically significant positive association (harmful effect) in the Hardell et al. studies (OR, 1.15—95% CI, 1.00 to 1.33— *n* = 10), a statistically significant negative association (beneficial effect) in the INTERPHONE-related studies (case-control studies from 13 countries coordinated by the International Agency for Research on Cancer (IARC); (OR, 0.81—95% CI, 0.75 to 0.89—*n* = 9), and no statistically significant association in other research groups’ studies. Further, cellular phone use with cumulative call time more than 1000 h statistically significantly increased the risk of tumors. This comprehensive meta-analysis of case-control studies found evidence that linked cellular phone use to increased tumor risk.

## 1. Introduction

According to estimates from the International Telecommunication Union, the number of worldwide mobile cellular subscriptions increased from 68.0 per 100 inhabitants in 2009 to 108.0 per 100 inhabitants in 2019 [1]. With the increasing use of cellular phones, concerns have arisen over the carcinogenic effects of electromagnetic fields (EMFs) emitted from cellular phones [2]. Since 1999, observational epidemiologic studies, specifically case-control studies have reported inconsistent findings on the association between cellular phone use and tumor risk, and several meta-analyses [3,4,5,6] of case-control studies on this topic have been published before 2011.

Among these studies, Myung et al.’s meta-analysis [5] of 23 case-control studies concluded that mobile phone use was associated with an increased tumor risk in high quality studies and studies conducted by a specific research group, and that long-term mobile phone use of 10 or more years increased the risk of tumors regardless of methodological quality or research group. Similarly, Khurana et al. also reported that cellular phone use of 10 or more years doubled the risk of brain tumors in 11 epidemiologic studies [6].

Based on evaluation of the available literature including experimental animal studies and epidemiological studies in humans, in 2011, the World Health Organization (WHO)/International Agency for Research on Cancer (IARC) classified radiofrequency electromagnetic fields (RF-EMFs) associated with cellular phone use as possibly carcinogenic to humans [7]. Recently, an advisory group of 29 scientists recommended that IARC prioritize a new review of the carcinogenicity of RF-EMF by 2024 due to mechanistic evidence of the carcinogenicity of cell phone radiation published since 2011 [8].

Although many case-control studies and several meta-analyses have been published regarding the association between cellular phone use and tumor risk, the findings remain inconsistent.

The purpose of this study was to evaluate the associations between cellular phone use and tumor risk using a systematic review and meta-analysis of case-control studies according to various factors including differences in response rates between cases and controls, use of blinding at interview for ascertainment of exposure, methodological quality, funding sources, type of case-control study, malignancy of tumor, and dose–response relationship.

## 2. Materials and Methods

### 2.1. Literature Search

We searched PubMed and EMBASE in July 2018, using common keywords related to cellular phones and tumors as follows: “cellular phone or mobile phone,” and “‘tumor or cancer”. We also located additional articles by reviewing the bibliographies of relevant articles.

### 2.2. Selection Criteria

We selected articles based upon the following criteria: case-control studies; investigated the associations between cellular phone or mobile phone use (not cordless phones) and the risk of benign or malignant tumors; reported outcome measures with adjusted odds ratios (OR) with 95% confidence intervals (CIs); and peer-reviewed articles written in English. If data were duplicated or shared in more than one article, we selected only the article with the larger sample size.

### 2.3. Selection of Relevant Studies

Two authors (Y.-J.C and Y.-R.L) independently reviewed the articles from the search and selected articles meeting the predetermined selection criteria. Disagreements between the two authors were resolved by discussion.

### 2.4. Assessment of Methodological Quality

We evaluated the methodological quality of the case-control studies based on the Newcastle-Ottawa Scale (NOS) [9] and the National Heart, Lung, and Blood Institute (NHLBI) quality assessment tool of case-control studies [10]. A star system of the NOS ranging from 0 to 9 is composed of three subscales: selection of study groups, comparability, and exposure. The NHLBI quality assessment tool consists of 12 questions answered with yes, no, or other (cannot determine, not applicable, or not reported). Two authors (Y.-J.C and Y.-R.L) independently assigned a score for each study, and disagreements were resolved by discussion. We considered a study awarded a number of stars or “yes” more than the mean of all the included studies as a high-quality study because standard criteria have not been established.

### 2.5. Main and Subgroup Analyses

We investigated the associations between cellular phone use (used vs. never or rarely used) and tumor risk by using adjusted data for the main analysis. When an individual study reported data on both analog and digital phones, the data on digital phones were selected. We also conducted subgroup meta-analyses by research group: Hardell et al. studies (Hardell studies), the INTERPHONE-related studies (INTERPHONE case-control studies in 13 countries coordinated by the International Agency for Research on Cancer [IARC]), and studies by other groups. Additionally, for each research group, we conducted subgroup meta-analyses by various factors as follows: difference in response rates between cases and controls (smaller difference vs. larger difference, by difference in response rates of 14.5%, which was an average difference in response rates between cases and controls in all studies), use of blinding at interview for ascertainment of exposure (used vs. not used or no description), methodological quality by the NOS (high vs. low, by average score), funding sources (cellular phone industry funding vs. not funded), type of case-control study (hospital-based vs. population-based), and malignancy of tumor (malignant vs. benign).

In order to evaluate an exposure–response relationship, we also performed subgroup meta-analyses by time since first use or latency (<5 vs. 5–9 vs. ≥10 years), cumulative or lifetime use (<5 vs. 5–9 vs. ≥10 years), cumulative call time (<300 vs. 300–1000 vs. ≥1000 h), and cumulative number of calls (<1000 vs. 1000–7000 vs. >7000). Latency refers to the length of time between the beginning of regular cellular phone use and the diagnosis of tumor occurrence. When multiple ORs with 95% CI were presented within each category of time or number of calls, a longer time or a higher number of calls was used for the analysis.

### 2.6. Statistical Analysis

To compute a pooled OR with its 95% CI, we used adjusted data from individual studies. A random-effects model meta-analysis on the basis of the DerSimonian and Laird method [11] was used in the current study because individual trials were carried out in the different populations. We also used a chi-square test to evaluate any differences in response rates between the case and control groups. We tested heterogeneity across the studies using Higgins I^2^, which represents the percentage of total variation within studies meta-analyzed [12]. I^2^ was calculated as below:

I^2^ = 100% × (Q − df)/Q, (1)

where Q is Cochran’s heterogeneity statistics, and df represents the degrees of freedom. Negative values of I^2^ are set to zero, and I^2^ lies between 0% (no observed heterogeneity) and 100% (maximal heterogeneity). We estimated publication bias using Begg’s funnel plot and Egger’s test. When there is publication bias, Begg’s funnel plot exhibits asymmetry, or the *p*-value < 0.05 by Egger’s test. The Stata SE version 14.0 software package was used for statistical analysis (StataCorp, College Station, TX, USA).

## 3. Results

### 3.1. Study Selection

**Figure 1:**
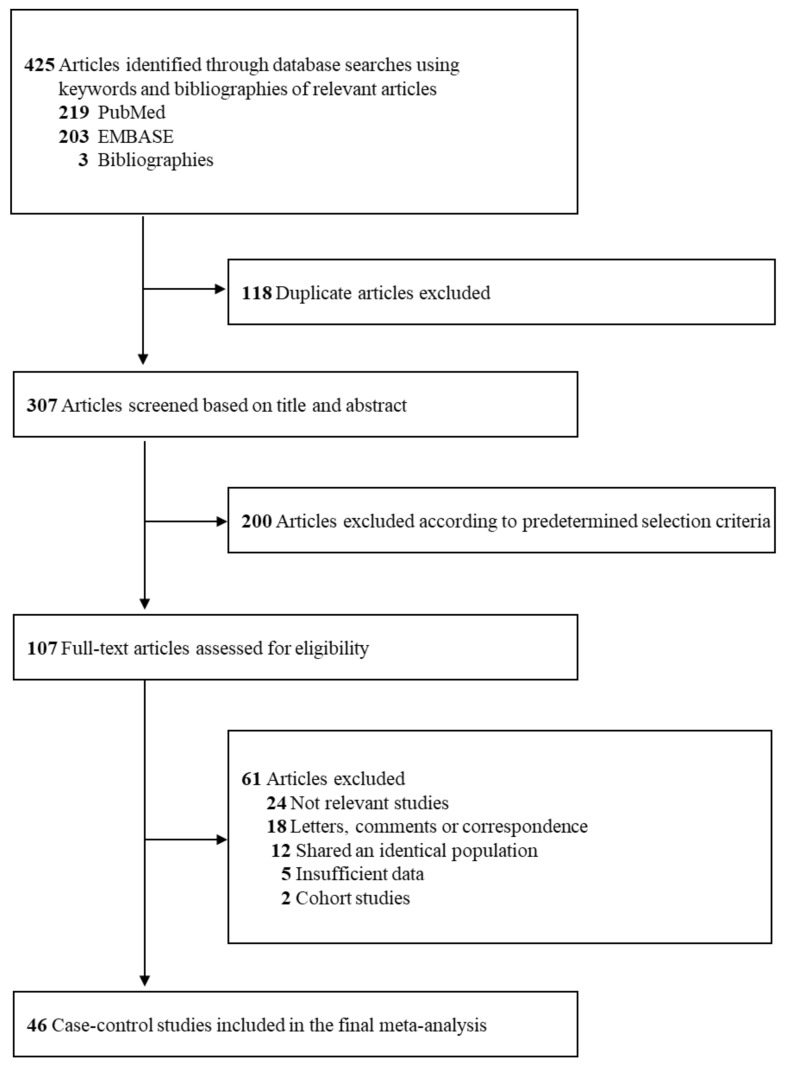
Study selection.

Figure 1 shows a flow diagram for the selection process of relevant studies. We identified a total of 425 articles from three core databases with 219 articles from PubMed, 203 articles from EMBASE, and 3 articles from hand-search. After excluding 118 duplicate articles and 200 articles that did not satisfy the pre-determined selection criteria by reviewing those titles and abstracts, the full texts of the remaining 107 articles were assessed for the final selection. After reviewing the full texts, 61 articles were excluded for the following reasons: not relevant studies (*n* = 24), letters, comments, or correspondence (*n* = 18), shared an identical population (*n* = 12), insufficient data (*n* = 5), and cohort studies (*n* = 2). The remaining 46 case-control studies (13–58) were included in the final analysis.

### 3.2. General Characteristics of Studies and Participants

General characteristics of the case-control studies included in the meta-analysis are shown in Table 1. The 46 case-control studies involved a total of 66,075 participants with 24,717 cases and 41,358 controls. For studies reporting gender, 53.9% of study participants were women. A total of 37 studies were hospital-based case-control studies, while nine studies were population-based case-control studies. The included studies were conducted in the following countries: Sweden (*n* = 24), Denmark (*n* = 9), United Kingdom (*n* = 8), Finland (*n* = 7), Norway (*n* = 6), Germany (*n* = 5), US (*n* = 4), Israel (*n* = 3), Japan (*n* = 2), Italy (*n* = 2), New Zealand (*n* = 2), France (*n* = 2), Brazil (*n* = 1), China (*n* = 1), South Korea (*n* = 1), and Thailand (*n* = 1). The most common type of tumor in the included studies was brain tumor (34 out of 46 studies, 74%), and the next most common ones were head and neck cancer such as parotid gland tumor (5/46, 12%), hematologic malignancies such as leukemia and non-Hodgkin’s lymphoma (4/46, 8.7%), melanoma (2/46, 4.3%), and testicular cancer (1/46, 2.2%).

The studies were classified by research group, i.e., Hardell studies (*n* = 11), INTERPHONE studies (*n* = 19), and studies conducted by other groups (*n* = 16). As shown in Table S1 and Table S2, the NOS scores ranged between 4 and 8 (average score, 6.4), and the NHLBI quality assessment scores ranged between 6 and 10 (average score, 8.3). We considered studies with an NOS score of ≥7 stars or an NHLBI quality assessment score of ≥9 points as having high quality and the remaining studies as having low quality.

The Hardell studies were not funded by the cellular phone industry. Most had high scores of ≥7 stars in the NOS and high scores of ≥9 points in the NHLBI quality assessment; most reported high response rates (>70%) with smaller differences in response rates (<14.5%) between the case group and the control group; and all were population-based case-control studies (Table 2, Table S1, and Table S2). All of the INTERPHONE studies were partly funded by the cellular phone industry (precisely, supported by funding from the International Union against Cancer, which received funds from the Mobile Manufacturers’ Forum and Global System for Mobile Communications Association) except for the INTERPHONE-Japan studies. Most had low scores of <7 stars and low scores of <9 points, showed low response rates (<70%), and had larger differences in response rates (>14.5%) between the case group and the control group. All were population-based case-control studies (Table 2, Table S1, and Table S2).

No study conducted by the other groups was funded by the cellular phone industry. Most of these studies had low response rates and mainly larger differences in response rates between the case group and the control group (Table 2).

### 3.3. Overall Use of Cellular Phone and Risk of Tumors

**Figure 2:**
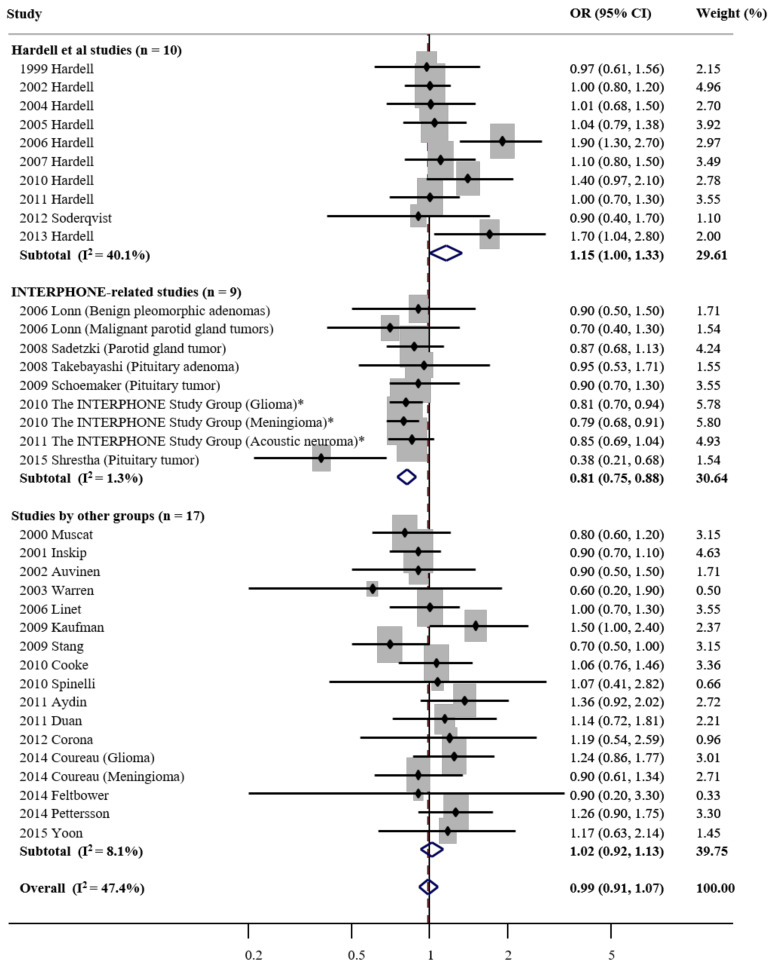
Cellular phone use and risk of tumors in a random-effects subgroup meta-analysis of case-control studies by research groups (*n* = 36). OR—odds ratio; CI—confidence interval. *—2010 and 2011 The INTERPHONE Study Group studies involved 13 countries.

As shown in Figure 2, as compared with never or none, the overall use of cellular phones was not associated with tumor risk in a random-effects meta-analysis of all 36 studies (OR, 0.99; 95% CI, 0.91 to 1.07; I^2^ = 47.4). Of the 46 studies, several [24,25,26,27,28,29,30,32,33,34,35,36] were excluded from the main analysis but included in the subgroup meta-analysis because study subjects overlapped with the INTERPHONE study published in 2010 [40] and 2011 [41] (which reported pooled results from all 13 countries).

In the subgroup meta-analysis by research group, cellular phone use was associated with marginally increased tumor risk in the Hardell studies (OR, 1.15 (95% CI, 1.00 to 1.33; *n* = 10; I^2^ = 40.1%), whereas it was associated with decreased tumor risk in the INTERPHONE studies (OR, 0.81; 95% CI, 0.75 to 0.88; *n* = 9; I^2^ = 1.3%). In the studies conducted by other groups, there was no statistically significant association between the cellular phone use and tumor risk (OR, 1.02; 95% CI, 0.92 to 1.13; *n* = 17; I^2^ = 8.1%).

Publication bias was not observed overall (Begg’s funnel plot was symmetric; Egger’s test, *p* for bias = 0.07). In addition, there was no publication bias in the subgroup meta-analysis by research group (Egger’s test, *p* for bias = 0.36 in the Hardell studies, 0.57 in the INTERPHONE studies, and 0.68 in studies by other groups, respectively).

### 3.4. Use of Cellular Phones and Risk of Tumors in Subgroup Meta-analysis By Various Factors

Table 2 shows the findings of the subgroup meta-analyses by various factors. Cellular phone use was statistically significantly associated with increased tumor risk in studies that used blinding at interview (OR, 1.16; 95% CI, 1.01 to 1.34; *n* = 10; I^2^ = 39.4%). In addition, cellular phone use had a marginally statistically significant association with increased tumor risk in studies with high methodological quality (OR, 1.11; 95% CI, 1.00 to 1.22; *n* = 17; I^2^ = 20.1%, based on the NOS score; OR, 1.09; 95% CI, 0.99 to 1.20; *n* = 20; I^2^ = 29.3, based on the NHLBI quality assessment tool). In contrast, cellular phone use had statistically significant associations with reduced tumor risk in studies that did not use blinding at interview, or were rated as having low methodological quality. Both the NOS score and NHLBI quality assessment tool showed similar findings in methodological quality scores: most Hardell studies were rated high quality, while most INTERPHONE studies were rated low quality.

Similarly, subgroup meta-analyses by funding source revealed a non-significant increased risk of tumors by cellular phone use in studies not funded by the cellular phone industry (OR, 1.07; 95% CI, 0.98 to 1.17; *n* = 28; I^2^ = 21.9%), whereas a statistically significantly decreased risk of tumors was observed in studies partly funded by the cellular phone industry (OR, 0.81; 95% CI, 0.74 to 0.89; *n* = 8; I^2^ = 0%), all of which were INTERPHONE studies.

Cellular phone use was not statistically significantly associated with tumor risk in the subgroup meta-analysis by type of case-control study. In the subgroup meta-analysis by type of tumor, a significantly decreased risk of benign tumors was observed (OR, 0.86; 95% CI, 0.77 to 0.95; *n* = 14; I^2^ = 21.9), while no significant association was observed for malignant tumors. This decreased risk of benign tumors was only found in INTERPHONE studies, not in Hardell et al. studies and studies by other groups.

### 3.5. Exposure–Response Relationship Between Use of Cellular Phones and Risk of Tumors

Table 3 shows an exposure-response relationship between cellular phone use and tumor risk. In the subgroup meta-analysis by time since first use or latency, overall the risk of tumors by cellular phone use non-significantly increased from an OR of 0.97 to 1.29 as latency increased from less than 5 years to 10 or more years. This finding was observed in each subgroup meta-analysis by research group. Especially, statistically significant increased tumor risk was observed for latency of 10 or more years in the Hardell studies (OR, 1.62; 1.03 to 2.57; *n* = 5; I^2^ = 39.9%). Similarly, the use of cellular phones non-significantly increased the risk of tumors as the cumulative or lifetime use in years and the cumulative number of calls increased in all studies and in each study group. Remarkably, in the subgroup meta-analysis of all studies by cumulative call time, cellular phone use greater than 1000 h statistically significantly increased the risk of tumors (OR, 1.60; 1.12 to 2.30; *n* = 8; I^2^ = 74.5%). Interestingly, the use of cellular phones overall and in the Hardell studies (OR, 3.65; 1.69 to 7.85; *n* = 2, especially in the Hardell studies) non significantly increased the risk of tumors with cumulative call time of 300–1000 h and more than 1000 h, while it decreased the risk of tumors in most subgroup meta-analyses of the INTERPHONE studies.

### 3.6. Use of Cellular Phones and Risk of Tumors in Subgroup Meta-analysis By Type of Tumor

Table S3 shows the findings from the subgroup meta-analyses by type of tumor. There was no statistically significant association between cellular phone use and tumor risk in most subgroup meta-analyses. Increased tumor risk was found for malignant brain tumors only in the Hardell studies (OR, 1.35; 95% CI, 1.06 to 1.73; *n* = 5; I^2^ = 53.9%).

## 4. Discussion

In this comprehensive systematic review and meta-analysis, we found statistically significant differences in the findings for the association between cellular phone use and tumor risk which varied by research group. Namely, there was a statistically significant increased association by 15% in the Hardell studies, a statistically significant decreased association by 19% in the INTERPHONE studies (multi-national case-control studies coordinated by the IARC), and no significant association in the other research groups’ studies. Importantly, in the subgroup meta-analysis of all studies reporting cumulative call times greater than 1000 h, cellular phone use with cumulative call time greater than 1000 h (about 17 min per day over a 10 year period) increased the risk of tumors by 60%.

Perhaps due to methodological deficiencies, cellular phone use appeared to reduce tumor risk in the INTERPHONE studies. These studies were partly funded by the mobile industry, had poor methodological quality, showed larger differences in response rates between the case and control groups, and did not use blinding at interview.

A substantial research literature documents potential mechanisms for the effects of cellular phone use on tumor risk. Although heating is the only biological effect of non-ionizing radiation (NIR) (including microwave radiation from cellular phones) recognized by most health agencies, numerous in vitro studies and animal studies demonstrated other possible mechanisms including increasing oxidative DNA damage and altering protein structure and expression [59]. In addition to a human endothelial cell line study, a human volunteer study reported a local exposure of human skin to RF-EMF caused changes in protein expression [60].

Based on the findings from pre-clinical studies, previous observational epidemiological studies, mainly case-control studies have reported inconsistent findings on the associations between cellular phone use and tumor risk. In 2009, we first reported evidence linking mobile phone use to increased tumor risk in a meta-analysis of low-biased case-control studies, especially among mobile phone users of 10 years or longer [5]. Two years later, the WHO/IARC classified RF-EMF due to cellular phone use as Group 2B, or “possibly carcinogenic to humans.” [7] Since then, subsequent case-control studies have reported inconsistent findings regarding the association between cellular phone use (use vs. never or rarely use) and tumor risk, similar to our previous findings. Since we published our meta-analysis in 2009, six meta-analyses [61,62,63,64,65,66] have reported the associations between cellular phone use and risk of brain tumors or head and neck tumors, mainly glioma and salivary gland tumors. Among them, four meta-analyses concluded that there was a statistically significant increased risk of glioma among heavy or long-term (over 10 years) mobile phone users in meta-analyses of 10 to 12 case-control studies [61,64,65,66]. In addition, one [62] of the remaining meta-analyses demonstrated a statistically significantly higher risk of all types of intracranial tumors in long-term mobile phone users (over 10 years) in a meta-analysis of 24 case-control studies, and the other [63] reported a statistically significantly increased risk of parotid gland tumors in a meta-analysis of three case-control studies.

Although the above mentioned four recent meta-analyses of case-control studies reported a significant increased risk of glioma in heavy or long-term (over 10 years) mobile phone users with an odds ratio of 1.35 in Wang et al. [61], 1.44 in Yang et al. [64], 1.33 in Wang et al. [65], and 1.33 in Prasad et al. [66], our study found a non-significantly increased risk with an OR of 1.66. This difference is due to the following reasons: Wang et al.’s meta-analysis in 2016 [61] reported that a significant association was found between mobile phone use of more than 5 years and glioma risk (OR = 1.35; 95% CI, 1.09 to 1.62; *p* < 0.05). However, when we reviewed the main results and Figure 1 in their article, the OR with 95% CI for mobile phone use of more than 5 years was 1.64 with 1.12 to 2.15. More importantly, when we performed a random-effects meta-analysis using the same data used in their analysis, there was no significant association between long-term use (>5 years) of mobile phones (the correct OR with 95% CI was 1.12 with 0.80 to 1.56). Yang et al.’s meta-analysis in 2017 [64] used seven studies comprising a Hardell study, a study by another group, and five INTERPHONE studies for long-term mobile phone use of 10 years or longer. The five INTERPHONE studies [26,27,29,30,34] were four publications [26,27,29,30] from individual countries (Denmark, Sweden, UK, and Germany) and one publication [34] of a collaborative analysis from five countries (Denmark, Finland, Norway, Sweden, and UK) within the same study years (2000–2004). Thus, Yang et al. used identical populations in three countries (Denmark, Sweden, and UK) in duplicates and used a smaller dataset from five countries instead of collaborative data [40] on glioma for the INTERPHONE studies from 13 countries published in 2010. When we performed a meta-analysis using the 2010′s collaborative data [40] instead of the five studies used in Yang et al.’s analysis, which were partly duplicated and smaller in sample size and number of countries than the 2010 collaborative analysis of the INTERPHONE group, there was no significant association between long-term mobile use and the risk of glioma (OR, 1.49; 95% CI, 0.80 to 2.78; *n* = 3; I^2^ = 91.5%), which is closer to our finding. Wang et al.’s meta-analysis in 2018 [65] included two cohort studies as well as case-control studies. More importantly, they included four ORs of >10–15 years, >15–20 years, >20–25 years, and >25 years from Hardell’s 2015 study [67]. If each OR is calculated from independent data (not overlapping), they can be combined. However, each reference used for the calculation of each OR was overlapping. When we conducted a meta-analysis using only an OR of 1.40 for 10–15 years of wireless phone use in Hardell’s 2015 study based on the Wang et al. analysis, there was no significant association between long-term use and the risk of glioma (OR, 1.08; 95% CI, 0.90 to 1.30; *n* = 6; I^2^ = 49.2%).

Compared to previous meta-analyses, the current meta-analysis has several strengths. First, the current meta-analysis is the most comprehensive study conducted to date, as it included 46 case-control studies with various types of tumors other than brain tumors. Second, we performed critical subgroup meta-analyses by factors that could affect individual results, such as the difference in response rates between cases and controls and funding sources, as well as use of blinding at interview for ascertainment of exposure and methodological quality. From these crucial subgroup meta-analyses, we confirmed that the opposite findings between the Hardell studies (increased tumor risk among cellular phone users) and the INTERPHONE studies (decreased tumor risk among cellular phone users) were closely associated with these factors. The INTERPHONE studies had differential response rates in case and control groups, did not use blinding at interview, had low methodological quality scores, and were partly funded by the cellular phone industry. In contrast, the Hardell studies had comparable response rates in case and control groups, used blinding at interview, had high methodological quality, and had no industry funding. Although there was no statistical significance, similar findings were observed in the subgroup meta-analysis by the above mentioned factors in the studies by other groups. In the current main analysis of 36 case-control studies, nine out of 10 Hardell studies showed smaller differences in response rates between case and control groups and had high response rates of about 80–90% in both groups. In contrast, all of the INTERPHONE studies showed larger differences in response rates between both groups; most had lower response rates in the control group than in the case group, and most had low response rates of about 40–70%. Over the past decades, participation rates (response rates in this study) have decreased in case-control studies, particularly in controls, which could lead to non-representative selection of controls, reducing the validity of the effect estimates, and casting doubt on the veracity of study findings [68]. Thus, the decreased risks of tumors observed in the INTERPHONE studies might be due to selection bias from participation of cellular phone users in the control group [69]. We also found that studies partly funded by the cellular phone industry showed a statistically significantly decreased risk of tumors by cellular phone use, all of which were INTERPHONE studies. It remains unclear whether cellular phone industry funding affected the study planning and conduct or data analysis and interpretation because the authors reported that the provision of funds to the study investigators via the UICC was governed by agreements that guaranteed INTERPHONE’s complete scientific independence. Nonetheless, many of these investigators rely upon industry for future research funding so they may have “hidden conflicts” of interest despite such agreements [70].

Our meta-analysis is based upon case-control studies which potentially suffer from recall bias and selection bias. Although prospective cohort studies typically enable stronger inferences to be drawn regarding causality, these studies are difficult to conduct when the outcome is a rare chronic disease that requires long-term exposure and subjects are exposed to multiple potential toxins. So far, two prospective cohort studies have been published [71,72]. Both employed inadequate measures of cell phone use, and one misclassified many cell phone users as non-users [71]. A large, international prospective cohort study is ongoing but will not yield results on tumor risk for 20 or more years [73].

There are several limitations in the current study. Although cordless phones often have a much higher power output than cellular phones, and the users of analogue phones have used longer than those of digital phones, we excluded the impact of those phones in this analysis. This might lead to a bias that underestimates the effect of mobile phones on the risk of cancer. In addition, we did not consider ipsilateral and contralateral use of the cellular phones, which is beyond the scope of our study. Lastly, although we reported exposure-response relationships between the cellular phone use and the cancer risk, it would be ideal to investigate those associations based on the actual time spent on cellular phones provided by the mobile telecommunication companies. However, most studies did not use those data. Further studies using the exact data on the time spent on cellular phones are warranted to confirm our findings.

## 5. Conclusions

In sum, the updated comprehensive meta-analysis of case-control studies found significant evidence linking cellular phone use to increased tumor risk, especially among cell phone users with cumulative cell phone use of 1000 or more hours in their lifetime (which corresponds to about 17 min per day over 10 years), and especially among studies that employed high quality methods. Further quality prospective studies providing higher level of evidence than case-control studies are warranted to confirm our findings.

## Figures and Tables

**Table 1:** General characteristics of studies included in the meta-analysis (*n* = 46).

Study ^a^	Country	Study Design ^b^	Study Period	Type of Tumor (Age Range, Years)	Type of Cellular Phone Used in Analysis	Exposure	OR (95% CI)	Adjusted Variables	No. (Response Rate)	
									Cases	Controls
**Hardell et al. studies (*n* = 11)**										
Hardell et al., 1999 [13]	Sweden	PCC	1994–1996	Brain tumor (20–80)	Digital	Use vs. no use (latency period >1 year)	0.97 (0.61 to 1.56)	Age, sex, and study region (matched)	209 (90%)	425 (91%)
Hardell et al., 2002 [14]	Sweden	PCC	1997–2000	Brain tumor (20–80)	Digital	Use vs. no use (latency period >1 year)	1.0 (0.8 to 1.2)	Use of different types of phones	1429 (88%)	1470 (91%)
Hardell et al., 2003 [15]	Sweden	PCC	1997–2000	Vestibular schwannoma (All ages)	Digital	Use vs. no use (latency period >1 year)	1.21 (0.66 to 2.22)	Sex, age, and geographical area	159 (89%)	159 (89%)
Hardell et al., 2004 [16]	Sweden	PCC	1994–2000	Salivary gland tumors (21–80)	Digital	Use vs. no use (latency period >1 year)	1.01 (0.68 to 1.50)	Age and sex	267 (91%)	1053 (90%)
Hardell et al., 2005 [17]	Sweden	PCC	1999–2002	Non-Hodgkin’s lymphoma (18–74)	Digital	Use vs. no use (latency period >1 year)	1.04 (0.79 to 1.38)	Age, sex, and year of diagnosis (cases) or enrollment (controls)	910 (91%)	1016 (92%)
Hardell et al., 2006 [18]	Sweden	PCC	2000–2003	Malignant brain tumor (20–80)	Digital	Use vs. no use (latency period >1 year)	1.9 (1.3 to 2.7)	Age, sex, socioeconomic index, and year of diagnosis	317 (88%)	692 (84%)
Hardell et al., 2007 [19]	Sweden	PCC	1993–1997	Testicular cancer (20–75)	Digital	Use vs. no use (latency period >1 year)	1.1 (0.8 to 1.5)	Age, year of diagnosis, and cryptorchidism	889 (91%)	870 (89%)
Hardell et al., 2010 [20]	Sweden	PCC	1997–2003	Malignant brain tumor (20–80)	Digital	Use vs. no use (latency period >1 year)	1.4 (0.97 to 2.1)	Age, sex, socio-economic index code, and year of diagnosis	346 (75%)	619 (67%)
Hardell et al., 2011 [21]	Sweden	PCC	2000–2003	Malignant melanoma (20–77)	Analog and digital	Use vs. no use (latency period >1 year)	1.0 (0.7 to 1.3)	Age, gender, and year of diagnosis	347 (82%)	1184 (80%)
Söderqvist et al., 2012 [22]	Sweden	PCC	2000–2003	Salivary gland tumor (22–80)	Digital	Use vs. no use (latency period >1 year)	0.9 (0.4 to 1.7)	Age, sex, year of diagnosis, and socio-economic index code	69 (88%)	262 (83%)
Hardell et al., 2013 [23]	Sweden	PCC	2007–2009	Malignant brain tumor (18–75)	Digital	Use vs. no use (latency period >1 year)	1.7 (1.04 to 2.8)	Age, gender, socio-economic index code, and year of diagnosis	593 (87%)	1368 (85%)
**INTERPHONE-related studies (*n* = 19)**										
Christensen et al., 2004 [24]	Denmark	PCC	2000–2002	Acoustic neuroma (20–69)	Cellular	Used regular vs. never or rarely used	0.90 (0.51 to 1.57)	Education level, marital status, use of hands-free devices, and region	107 (82%)	214 (64%)
Lönn et al., 2004 [25]	Sweden	PCC	1999–2002	Acoustic neuroma (20–69)	Digital	Regular use vs. Never or rarely	0.9 (0.6 to 1.4)	Age, sex, residential area, and education	148 (93%)	604 (72%)
Christensen et al., 2005 [26]	Denmark	PCC	2000–2002	Low- grade glioma (20–69)	Cellular	Regular use vs. no regular use	0.58 (0.37 to 0.90)	Sex, age, education, hands-free devices in cars, marital status, and region	171 (74%)	330 (64%)
				High-grade glioma (20–69)			1.08 (0.58 to 2.00)		81 (74%)	155 (64%)
				Meningioma (20–69)			0.83 (0.54 to 1.28)		175 (74%)	316 (64%)
Lönn et al., 2005 [27]	Sweden	PCC	2000–2002	Glioma (20–69)	Digital	Regular use vs. never or rarely use	0.8 (0.6 to 1.0)	Age, gender, geographic region, and education	371 (74%)	674 (71%)
				Meningioma (20–69)			0.6 (0.5 to 0.9)			
Schoemaker et al., 2005 [28]	Denmark, Finland, Norway, Sweden, and UK	PCC	1999–2004	Acoustic neuroma (18–69)	Digital	Regular use vs. Never/non-regular use	0.9 (0.7 to 1.1)	Highest educational level and combinations of interview year and interview lag time	678 (83%)	3553 (51%)
Hepworth et al., 2006 [29]	UK	PCC	2000–2003	Glioma (18–69)	Mobile	Regular use vs. never/non-regular	0.94 (0.78 to 1.13)	Age, sex, region, Townsend deprivation category, and interview reference date category	966 (51%)	1716 (45%)
Schuz et al., 2006 [30]	Germany	PCC	2000–2003	Glioma (30–69)	Cellular	Ever use vs. never use	0.98 (0.74 to 1.29)	Age, socioeconomic status, and living in a city	366 (80%)	732 (60%)
				Meningioma (30–69)			0.84 (0.62 to 1.13)		381 (80%)	762 (60%)
Lönn et al., 2006 [31]	Denmark and Sweden	PCC	2000–2002	Benign pleomorphic adenomas (20–69)	Mobile	Regular use vs. never or rarely use	0.9 (0.5 to 1.5)	Age, gender, geographic region, and education	112 (88%)	321 (70%)
				Malignant parotid gland tumors (20–69)			0.7 (0.4 to 1.3)		60 (85%)	681 (70%)
Takebayashi et al., 2006 [32]	Japan	PCC	2000–2004	Acoustic neuroma (30–69)	Digital	Regular user vs. non-user	0.68 (0.40 to 1.18)	Education and marital status	101 (84%)	339 (52%)
Klaeboe et al., 2007 [33]	Norway	PCC	2001–2002	Glioma (19–69)	Digital	Regular use vs. no or irregular use	0.6 (0.4 to 0.8)	Age, sex, residential area, and education	289 (77%)	358 (69%)
				Meningioma (19–69)			0.6 (0.4 to 1.0)			
Lahkola et al., 2007 [34]	Denmark, Finland, Norway, Sweden, and UK	PCC	2000–2004	Glioma (20–69)	Digital	Regular use vs. never /non- regular use	0.75 (0.65 to 0.87)	None (adjustment for education and family history of glioma did not affect the result)	1521 (60%)	3301 (50%)
Schlehofer et al., 2007 [35]	Germany	PCC	2000–2003	Acoustic neuroma (30–69)	Mobile	Ever use vs. never use	0.67 (0.38 to 1.19)	SES, living area, age at diagnosis, and study center	97 (89%)	194 (55%)
Lahkola et al., 2008 [36]	Denmark, Finland, Norway, Sweden, and UK	PCC	2000–2004	Meningioma (18–69)	Digital	Regular use vs. never/non-regular	0.74 (0.63 to 0.87)	Sex, five-year age group, region, and country	1209 (74%)	3299 (50%)
Sadetzki et al., 2008 [37]	Israel	PCC	2001–2003	Parotid gland tumors (≥18)	Cellular	Regular user vs. no regular user (<1 year)	0.87 (0.68 to 1.13)	None (adjustment for cigarette smoking did not affect the result)	460 (87%)	1266 (66%)
Takebayashi et al., 2008 [38]	Japan	PCC	2000–2004	Glioma (30–69)	Digital	Regular user vs. non-user	1.29 (0.66 to 2.53)	Education and marital status	88 (59%)	196 (53%)
				Meningioma (30–69)			0.67 (0.40 to 1.13)		132 (78%)	279 (52%)
				Pituitary adenoma (30–69)			0.95 (0.53 to 1.71)		102 (76%)	208 (49%)
Schoemaker et al., 2009 [39]	UK	PCC	2001–2005	Pituitary tumor (18–59)	Digital	Regular use vs. never/non-regular use	0.9 (0.7 to 1.3)	Sex, age category, geographic area within study region, reference date, and Townsend deprivation score	291 (63%)	630 (43%)
The INTERPHONE Study Group, 2010 [40]	13 ^c^ Countries	PCC	2000–2004	Glioma (30–59)	Mobile	Regular use vs. no regular use	0.81 (0.70 to 0.94)	Sex, age, study center, ethnicity in Israel, and education	2708 (64%)	2972 (53%)
				Meningioma (30–59)			0.79 (0.68 to 0.91)		2409 (78%)	2662 (53%)
The INTERPHONE Study Group, 2011 [41]	13 ^c^ Countries	PCC	2000–2004	Acoustic neuroma (30–59)	Mobile	Regular use vs. no regular use	0.85 (0.69 to 1.04)	Sex, age, study center, ethnicity, and education	1105 (85%)	2145 (53%)
Shrestha et al., 2015 [42]	Finland	PCC	2000–2002	Pituitary tumor (20–69)	Digital	Regular use vs. never /non-regular use	0.38 (0.21 to 0.68)	Not described	80 (42%)	240 (77%)
**Studies by other groups (*n* = 16)**										
Muscat et al., 2000 [43]	US	HCC	1994–1998	Brain cancer (18–80)	Cellular	Regular use vs. no use	0.8 (0.6 to 1.2)	Age, education, sex, race, study center, proxy subject, and month and year of interview	469 (82%)	346 (90%)
Inskip et al., 2001 [44]	US	HCC	1994–1998	Brain tumor (≥18)	Cellular	Use vs. no use	0.9 (0.7 to 1.1)	Age, sex, race, hospital, distance from patient’s residence to hospital, education, household income, date of interview, and interview respondent	782 (80%)	799 (86%)
Auvinen et al., 2002 [45]	Finland	PCC	1996	Brain tumor (20–69)	Digital	Ever use vs. never use	0.9 (0.5 to 1.5)	Described that adjusted odds ratios were calculated, and potential confounding factors were urban residence, socioeconomic status, and occupation	398 (n.a.)	2160 (n.a.)
Warren et al., 2003 [46]	US	HCC	1995–2000	Infratemporal facial nerve tumor (mean 47)	Cellular	Use vs. no use	0.6 (0.2 to 1.9)	Described that a multivariate model was used, but not presented	18 (n.a.)	141 (n.a.)
Linet et al., 2006 [47]	US	PCC	1998–2000	Non-Hodgkin’s lymphoma (20–74)	Cellular	Ever used vs. ever used	1.0 (0.7 to 1.3)	Age, ethnic group, education, and geographic site	551 (79%)	462 (55%)
Kaufman et al., 2009 [48]	Thailand	HCC	1997–2003	Leukemia (≥18)	Cellular	Use vs. no use	1.5 (1.0 to 2.4)	Age, sex, income, use of cellphones, benzene and other solvent exposure, occupational and non-occupational pesticide exposure, pesticides used near the home, working with power lines, and living near power lines	180 (n.a.)	756 (n.a.)
Stang et al., 2009 [49]	Germany	HCC	2002–2004	Uveal melanoma (20–74)	Mobile	Regular use vs. never	0.7 (0.5 to 1.0)	Age, sex, and residence	827 (94%)	455 (57%)
Cooke et al., 2010 [50]	UK	PCC	2003–2009	Leukemia (18–59)	Mobile	Regular use vs. never/non-regular use	1.06 (0.76 to 1.46)	Age, sex, socio-economic status, area of residence, ethnicity, smoking status, and interview lag time/period	806 (50%)	589 (75%)
Spinelli et al., 2010 [51]	France	HCC	2005	Brain cancer (20–87)	Cellular	>36 h-years vs. no use	1.07 (0.41 to 2.82)	Age and sex	116 (75%)	116 (90%)
Aydin et al., 2011 [52]	Denmark, Norway, Sweden, and Switzerland	PCC	2004–2008	Brain tumors (7–19)	Mobile	Regular use vs. no regular use	1.36 (0.92 to 2.02)	Unadjusted (SES, family history of cancer, past medical radiation exposure to the head, maternal smoking during pregnancy, past head injuries, and use of baby monitors did not change the results)	352 (83%)	646 (71%)
Duan et al., 2011 [53]	China	HCC	1993–2000	Epithelial parotid gland malignancies (7–80)	Cellular	Regular use vs. never or rarely use	1.14 (0.72 to 1.81)	Gender, age, resident area, marital status, education background, monthly income, and smoking status	136 (62%)	2051 (78%)
Corona et al., 2012 [54]	Brazil	HCC	2000–2010	Vestibular schwannoma (mean 49 in cases, 53 in controls)	Cellular	Regular use vs. no use/irregular use	1.19 (0.54 to 2.59)	Not described	44 (52%)	104 (57%)
Coureau et al., 2014 [55]	France	PCC	2004–2006	Glioma (≥16)	Mobile	Regular user vs. no regular user	1.24 (0.86 to 1.77)	Education and ionizing radiation exposure	253 (66%)	504 (45%)
				Meningioma (≥16)			0.90 (0.61 to 1.34)		194 (75%)	388 (45%)
Feltbower et al., 2014 [56]	UK	PCC	2007–2010	Brain tumor (0–24)	Mobile	Spoken on a mobile phone more than 20 times vs. not	0.9 (0.2 to 3.3)	Age, sex, and Townsend deprivation index	49 (52%)	78 (32%)
Pettersson et al., 2014 [57]	Sweden	PCC	2002–2007	Acoustic neuroma (20–69)	Digital	Regular use vs. never or rarely use	1.26 (0.90 to 1.75)	Unadjusted (smoking, education, marital status, parity, and hands-free use did not affect the results)	422 (83%)	643 (65%)
Yoon et al., 2015 [58]	Korea	HCC	2002–2007	Glioma (15–69)	Mobile	User vs. non-user	1.17 (0.63 to 2.14)	Age, sex, area, education, respondent type, hair coloring, alcohol drinking, computer use, and electro-blanket use	285 (32%)	285 (27%)

^a^ Numbers in parentheses indicate the reference numbers in the full text. ^b^ HCC, hospital-based case-control studies; PCC, population-based case-control studies. ^c^ Australia, Canada, Denmark, Finland, France, Germany, Israel, Italy, Japan, New Zealand, Norway, Sweden, and UK. n.a.: not available.

**Table 2:** Use of cellular phones and risk of tumors in subgroup meta-analysis of case-control studies.

Factor			All			Hardell et al. Studies			INTERPHONE-Related Studies			Studies by Other Groups		
			No.	OR (95% CI)	I^2^ (%)	No.	OR (95% CI)	I^2^ (%)	No.	OR (95% CI)	I^2^ (%)	No.	OR (95% CI)	I^2^ (%)
			36	0.99 (0.91 to 1.07)	47.4	10	1.15 (1.00 to 1.33) *	40.1	9	0.81 (0.75 to 0.88)	1.3	17	1.02 (0.92 to 1.13)	8.1
**Difference in response rates ^a^**	Smaller (<14.5%)		16	1.07 (0.94 to 1.21)	54.2	10	1.15 (1.00 to 1.33) *	40.1	1	0.81 (0.70 to 0.94)	*n*.a.	5	0.99 (0.81 to 1.2)	21.1
	Larger (>14.5%)		17	0.91 (0.82 to 1.02)	23.8	n.a.			8	0.81 (0.73 to 0.91)	13.7	9	1.02 (0.90 to 1.17)	0.0
**Use of blinding at interview**	Used		10	1.16 (1.01to 1.34) *	39.4	9	1.16 (1.00 to 1.35) *	45.4	n.a.			1	1.19 (0.54 to 2.59)	n.a.
	Not used		26	0.91 (0.84 to 0.99)	32.1	1	0.90 (0.44 to 1.70)	n.a.	9	0.81 (0.75 to 0.88)	1.3	16	1.02 (0.91 to 1.13)	13.0
**Methodolog-ical quality ^b^**	High	NOS	17	1.11 (1.00 to 1.22) *	20.1	9	1.16 (1.00 to 1.35) *	45.4	1	0.90 (0.66 to 1.23)	n.a.	7	1.08 (0.92 to 1.27)	0.0
		NHLBI	20	1.09 (0.99 to 1.20)	29.3	8	1.18 (1.00 to 1.40)	50.7	2	0.80 (0.54 to 1.20)	0.0	10	1.03 (0.91 to 1.15)	0.0
	Low	NOS	19	0.88 (0.80 to 0.97)	33.9	1	0.90 (0.44 to 1.70)	n.a.	8	0.81 (0.74 to 0.88)	8.5	10	0.99 (0.85 to 1.16)	30.5
		NHLBI	16	0.86 (0.78 to 0.95)	27.2	2	0.95 (0.64 to 1.41)	0.0	7	0.81 (0.74 to 0.90)	22.4	7	0.99 (0.79 to 1.24)	31.2
**Funding by cellular phone industry**	Not funded		28	1.07 (0.98 to 1.17)	21.9	10	1.15 (1.00 to 1.33) *	40.1	1	0.95 (0.53 to 1.71)	n.a.	17	1.02 (0.92 to 1.13)	8.1
	Funded		8	0.81 (0.74 to 0.89)	10.6	n.a.			8	0.81 (0.74 to 0.89)	10.6	n.a.		
**Type of case-control study**	HCC		9	0.95 (0.80 to 1.12)	22.4	n.a.			n.a.			9	0.95 (0.80 to 1.12)	22.4
	PCC		27	1.00 (0.91 to 1.09)	53.7	10	1.15 (1.00 to 1.33) *	40.1	9	0.81 (0.75 to 0.88)	1.3	8	1.10 (0.96 to 1.26)	0.0
**Malignancy**	Malignant		21	1.08 (0.97 to 1.20)	31.4	9	1.18 (1.02 to 1.37)	38.5	2	0.84 (0.54 to 1.31)	0.0	10	0.97 (0.84 to 1.12)	8.8
	Benign		14	0.86 (0.77 to 0.95)	21.9	3	0.92 (0.74 to 1.14)	38.6	8	0.81 (0.72 to 0.90)	14.6	3	1.07 (0.83 to 1.39)	4.3

^a^ A difference in response rates between cases and controls was measured based on the average difference in response rates of 14.5% points between cases and controls when combining all the studies. Three studies [51,52,54] did not report response rates; ^b^ The methodological quality of each study was assessed by the Newcastle-Ottawa Scale (NOS) and the National Heart, Lung, and Blood Institute (NHLBI) quality assessment tool of case-control studies. The NOS score of ≥7 stars or the NHLBI score of ≥9 were considered as having high quality, and that of <7 stars and that of <9 were considered as having low quality; No.,number of studies; n.a., not available; HCC, hospital-based case-control study; PCC, population-based case-control study; ‘*’ indicates that cellular phone use statistically significantly increases the risk of tumor.

**Table 3:** Exposure–response relationship between use of cellular phones and risk of tumors.

Factor		All			Hardell et al.’s Studies			INTERPHONE-Related Studies			Other Groups		
		No.	OR (95% CI)	I^2^	No.	OR (95% CI)	I^2^	No.	OR (95% CI)	I^2^	No.	OR (95% CI)	I^2^
**Time since first use or latency (years)**	**<5**	25	0.97 (0.86 to 1.09)	39.0	10	1.05 (0.92 to 1.19)	0.0	8	0.78 (0.64 to 0.94)	36.2	8	1.10 (0.92 to 1.32)	14.6
	**5–9**	23	1.00 (0.86 to 1.16)	51.0	10	1.20 (0.88 to 1.63)	44.4	8	0.80 (0.70 to 0.92)	13.7	5	1.19 (0.99 to 1.44)	0.0
	**≥10**	18	1.29 (0.90 to 1.85)	87.8	5	1.62 (1.03 to 2.57) *	39.9	8	0.99 (0.79 to 1.24)	25.3	5	1.57 (0.72 to 3.42)	93.3
**Cumulative or lifetime use (years)**	**<5**	14	0.81 (0.74 to 0.90)	19.6	n.a.			9	0.77 (0.69 to 0.86)	15.8	5	0.99 (0.81 to 1.21)	0.0
	**5–9**	14	0.89 (0.78 to 1.01)	22.9				9	0.83 (0.73 to 0.94)	0.0	5	1.04 (0.75 to 1.46)	54.4
	**≥10**	9	1.04 (0.69 to 1.59)	36.9				5	0.92 (0.54 to 1.59)	0.0	5	1.15 (0.61 to 2.18)	77.1
**Cumulative call time (hours)**	**<300**	26	0.99 (0.90 to 1.08)	0.0	9	1.08 (0.94 to 1.23)	9.2	9	0.78 (0.66 to 0.93)	0.0	8	1.05 (0.89 to 1.24)	0.0
	**300–1000**	7	1.14 (0.91 to 1.41)	40.9	1	1.00 (0.40 to 2.60)		2	1.07 (0.77 to 1.49)	0.0	4	1.21 (0.79 to 1.84)	40.9
	**>1000**	8	1.60 (1.12 to 2.30) *	74.5	2	3.65 (1.69 to 7.85) *	0.0	4	1.25 (0.96 to 1.62)	23.7	2	1.73 (0.66 to 4.48)	91.8
**Cumulative number of calls**	**<1000**	7	1.07 (0.87 to 1.32)	9.6	n.a.			2	0.70 (0.38 to 1.29)	0.0	5	1.13 (0.92 to 1.39)	0.0
	**1000–7000**	5	1.00 (0.69 to 1.43)	51.6	n.a.			n.a.			5	1.00 (0.69 to 1.43)	51.6
	**>7000**	10	1.14 (0.39 to 3.32)	98.6	n.a.			5	0.85 (0.56 to 1.29)	55.1	5	1.68 (0.36 to 7.94)	99.0

No.,number of studies; n.a., not available. ‘*’ indicates that cellular phone use statistically significantly increases the risk of tumor.

## Supplementary Materials

The following are available online at https://www.mdpi.com/1660-4601/17/21/8079/s1, Table S1: Methodological quality of case-control studies based on the Newcastle-Ottawa Scale (*n* = 46), Table S2: Methodological quality of case-control studies based on the National Heart, Lung, and Blood Institute quality assessment tool of case-control studies (*n* = 46), Table S3: Cellular phone use and risk of tumors in subgroup meta-analysis by type of tumor.
