# Supplementary material for: Cellular Phone Use and Risk of Tumors: Systematic Review and Meta-Analysis
Source: Int J Environ Res Public Health. 2020 Nov 2;17(21):8079. doi: 10.3390/ijerph17218079 (PMC7663653; doi:10.3390/ijerph17218079)
Supplement: Supplementary file 1 [file ijerph-17-08079-s001.pdf]

Table S1. Methodological Quality of Case-Control Studies Based on the Newcastle-Ottawa Scale (n = 46).

Study Name	Selection				Comparability	Exposure			Outcome
	**Adequate Definition of Patient Cases**	**Representativeness of Patient Cases**	**Selection of Controls**	**Definition of Controls**	**Control for Important Factor or Additional Factor**	**Assessment of Exposure (Blinding)**	**Same Method of Ascertainment for Participants**	**Non-response Rate**	
Hardell et al.'s studies									
Hardell et al., 1999 [13]	1	1	1	0	2	1	1	1	8
Hardell et al., 2002 [14]	0	1	1	0	2	1	1	1	7
Hardell et al., 2003 [15]	1	1	1	0	2	0	1	0	6
Hardell et al., 2004 [16]	0	1	1	0	2	1	1	1	7
Hardell et al., 2005 [17]	1	1	1	0	2	0	1	1	7
Hardell et al., 2006 [18]	0	1	1	0	2	1	1	1	7
Hardell et al., 2007 [19]	0	1	1	0	2	1	1	1	7
Hardell et al., 2010 [20]	0	1	1	1	2	0	1	1	7
Hardell et al., 2011 [21]	0	1	1	0	2	1	1	1	7
Soderqvist et al., 2012 [22]	1	1	1	0	2	0	1	1	7
Hardell et al., 2013 [23]	0	1	1	0	2	1	1	1	7

**INTERPHONE-related studies**									
Christensen et al., 2004 [24]	1	0	1	1	2	0	1	0	6
Lönn et al., [25] 2004	1	1	1	0	2	0	1	0	6
Christensen et al., 2005 [26]	1	1	1	1	2	0	1	0	7
Lönn et al., 2005 [27]	1	1	1	0	2	0	1	0	6
Schoemaker et al., 2005 [28]	1	1	1	1	2	0	1	0	7
Hepworth et al., 2006 [29]	1	1	1	1	2	1	0	1	8
Schuz et al., 2006 [30]	1	1	1	0	2	0	1	0	6
Lönn et al., 2006 [31]	1	1	1	0	2	0	1	0	6
Takebayashi et al., 2006 [32]	1	1	1	0	2	0	1	1	7
Klaeboe et al., 2007 [33]	1	1	1	1	2	0	1	1	8
Lahkola et al., 2007 [34]	1	1	1	0	2	0	1	0	6
Schlehofer et al., 2007 [35]	1	1	1	0	2	0	1	0	6
Lahkola et al., 2008 [36]	1	1	1	0	2	0	1	0	6
Sadetzki et al., [37] 2008	1	1	1	0	1	0	1	0	5
Takebayashi et al., 2008 [38]	1	1	1	0	2	0	1	0	6

Schoemaker et al., 2009 [39]	1	1	1	1	2	0	1	0	7
The INTERPHONE Study Group, 2010 [40]	1	1	1	0	2	1	1	0	7
The INTERPHONE Study Group, 2011 [41]	1	1	1	0	2	0	1	0	6
Shrestha et al., 2015 [42]	1	1	1	0	1	0	1	0	5
**Studies by other groups**									
Muscat et al., 2000 [43]	1	1	0	1	2	0	1	1	7
Inskip et al., 2001 [44]	1	1	0	0	2	0	1	1	6
Auvinen et al., 2002 [45]	0	1	1	0	2	1	1	0	6
Warren et al., 2003 [46]	1	0	1	0	2	1	1	0	6
Linet et al., 2006 [47]	1	1	1	1	2	0	1	0	7
Kaufman et al., 2009 [48]	1	0	0	0	2	1	1	0	6
Stang et al., 2009 [49]	1	0	1	0	2	0	1	0	5
Cooke et al., 2010 [50]	1	1	0	1	2	0	1	0	6
Spinelli et al., 2010 [51]	1	1	0	1	1	0	1	0	5

Aydin et al., 2011 [52]	1	1	1	0	2	1	1	0	7
Duan et al., 2011 [53]	1	0	0	1	2	0	0	0	4
Corona et al., 2012 [54]	1	1	0	1	0	1	1	1	6
Coureau et al., 2014 [55]	0	1	1	1	2	0	1	0	6
Feltbower et al., 2014 [56]	1	1	1	1	2	0	1	0	7
Pettersson et al., 2014 [57]	1	1	1	0	2	0	1	0	6
Yoon et al., 2015 [58]	1	1	0	1	2	0	1	1	7

Each study is awarded a maximum of one star for each item within the selection and exposure categories, while a maximum of two stars can be given for the comparability category.

Table 2. Methodological Quality of Case-control Studies Based on the National Heart, Lung, and Blood Institute quality assessment tool of case-control studies (n = 46).

**Study Name**	**Research Question**	**Study Population**	**Sample Size Justification**	**Groups Recruited from the Same Population**	**Selection Criteria**	**Case and control definitions**	**Random selection of study participants**	**Concurrent controls**	**Exposure assessed prior to outcome measurement**	**Exposure measures and assessment**	**Blinding of exposure assessors**	**Statistical analysis**	**No. of 'Yes'**
**Hardell et al.'s studies**													
Hardell et al., 1999 [13]	Y	Y	NA	Y	Y	NR	NR	Y	Y	Y	Y	N	**8**
Hardell et al.,[14)] 2002	Y	Y	NA	Y	Y	NR	Y	Y	Y	Y	Y	Y	**10**
Hardell et al., 2003 [15]	Y	Y	NA	Y	Y	NR	Y	NR	Y	Y	Y	Y	**9**
Hardell et al., 2004 [16]	Y	Y	NA	Y	Y	NR	Y	Y	Y	Y	Y	Y	**10**
Hardell et al., 2005 [17]	Y	Y	NA	Y	Y	NR	Y	Y	Y	Y	Y	Y	**10**
Hardell et al., 2006 [18]	Y	Y	NA	Y	Y	NR	Y	Y	Y	Y	Y	Y	**10**
Hardell et al., 2007 [19]	Y	Y	NA	Y	Y	NR	Y	Y	Y	Y	Y	Y	**10**
Hardell et al., 2010 [20]	Y	Y	NA	Y	Y	Y	NR	Y	Y	Y	NR	Y	**9**
Hardell et al., 2011 [21]	Y	Y	NA	Y	Y	NR	NR	Y	Y	Y	Y	Y	**9**

Soderqvist et al., 2012 [22]	Y	Y	NA	Y	Y	NR	Y	NR	Y	Y	NR	N	**7**
Hardell et al., 2013 [23]	Y	Y	NA	Y	Y	NR	Y	Y	Y	Y	Y	Y	**10**
**INTERPHONE-related studies**													
Christensen et al., 2004 [24]	Y	Y	NA	Y	Y	Y	Y	NR	Y	Y	NR	Y	**9**
Lönn et al., 2004 [25]	Y	Y	NA	Y	Y	NR	Y	Y	Y	Y	NR	Y	**9**
Christensen et al., 2005 [26]	Y	Y	NA	Y	Y	Y	Y	Y	Y	Y	NR	Y	**10**
Lönn et al., 2005 [27]	Y	Y	NA	Y	Y	NR	Y	Y	Y	Y	NR	Y	**9**
Schoemaker et al., 2005 [28]	Y	Y	NA	Y	Y	Y	Y	NR	Y	Y	NR	Y	**9**
Hepworth et al., 2006 [29]	Y	Y	NA	N	Y	NR	Y	NR	Y	Y	NR	Y	**7**
Schuz et al., 2006 [30]	Y	Y	NA	Y	Y	NR	Y	NR	Y	Y	NR	Y	**8**
Lönn et al., 2006 [31]	Y	Y	NA	Y	Y	NR	Y	Y	Y	Y	NR	Y	**9**
Takebayashi et al., 2006 [32]	Y	Y	NA	N	Y	NR	Y	NR	Y	Y	NR	Y	**7**

Klaeboe et al., 2007 [33]	Y	Y	NA	Y	Y	Y	Y	NO	Y	Y	NR	Y	**9**
Lahkola et al., 2007 [34]	Y	Y	NA	NA	Y	NA	Y	NR	Y	Y	NR	Y	**7**
Schlehofer et al., 2007 [35]	Y	Y	NA	N	Y	NR	Y	NR	Y	Y	NR	Y	**7**
Lahkola et al., 2008 [36]	Y	Y	NA	Y	Y	NA	Y	NR	Y	Y	NR	Y	**8**
Sadetzki et al., 2008 [37]	Y	Y	NA	Y	Y	NR	Y	NR	Y	Y	NR	Y	**8**
Takebayashi et al., 2008 [38]	Y	Y	NA	N	Y	NR	Y	NR	Y	Y	NR	Y	**7**
Schoemaker et al., 2009 [39]	Y	Y	NA	N	Y	Y	CD	NR	Y	Y	NR	Y	**7**
The INTERPHONE Study Group, 2010 [40]	Y	Y	NA	N	Y	NA	NA	NR	Y	Y	NR	Y	**6**
The INTERPHONE Study Group, 2011 [41]	Y	Y	NA	N	Y	NA	NA	NR	Y	Y	NR	Y	**6**
Shrestha et al., 2015 [42]	Y	Y	NA	Y	Y	NR	NR	NR	Y	Y	NR	Y	**7**

**Studies by other groups**												
Muscat et al., 2000 [43]	Y	Y	NA	Y	Y	Y	N	Y	Y	Y	NR	Y	**9**
Inskip et al., 2001 [44]	Y	Y	NA	Y	Y	Y	NR	Y	Y	Y	NR	Y	**9**
Auvinen et al., 2002 [45]	Y	Y	NA	Y	Y	Y	NR	NR	Y	Y	NR	Y	**8**
Warren et al., 2003 [46]	Y	Y	NA	Y	Y	Y	NR	NR	Y	Y	NR	Y	**8**
Linet et al., 2006 [47]	Y	Y	NA	Y	Y	Y	Y	NR	Y	Y	NR	Y	**9**
Kaufman et al., 2009 [48]	Y	Y	NA	CD	Y	Y	NR	NR	Y	Y	NR	Y	**7**
Stang et al., 2009 [49]	Y	Y	NA	CD	Y	Y	NR	NR	Y	Y	NR	Y	**7**
Cooke et al., 2010 [50]	Y	Y	NA	N	Y	Y	N	NR	Y	Y	NR	Y	**7**
Spinelli et al., 2010 [51]	Y	Y	NA	Y	Y	Y	Y	NR	Y	Y	NR	Y	**9**
Aydin et al., 2011 [52]	Y	Y	NA	Y	Y	NR	Y	Y	Y	Y	NR	Y	**9**
Duan et al., 2011 [53]	Y	Y	NA	Y	Y	Y	NR	NR	Y	Y	NR	Y	**8**

Corona et al., 2012 [54]	Y	Y	NA	Y	Y	Y	NR	Y	Y	Y	NR	Y	**9**
Coureau et al., 2014 [55]	Y	Y	NA	Y	Y	Y	Y	NR	Y	Y	NR	Y	**9**
Feltbower et al., 2014 [56]	Y	Y	NA	CD	Y	NR	Y	NR	Y	Y	NR	Y	**7**
Pettersson et al., 2014 [57]	Y	Y	NA	Y	Y	CD	Y	Y	Y	Y	NR	Y	**9**
Yoon et al., 2015 [58]	Y	Y	NA	Y	Y	Y	Y	NR	Y	Y	NR	Y	**9**

Y, yes; N, no; NA, not applicable; NR, not reported; CD, cannot determine.

Table S3. Cellular Phone Use and Risk of Tumors in Subgroup Meta-analysis by Type of Tumor.

		All			Hardell et al.'s Studies			INTERPHONE-Related studies			Studies by Other Groups		
		**No**	**OR (95% CI)**	**I² (%)**	**No**	**OR (95% CI)**	**I² (%)**	**No**	**OR (95% CI)**	**I² (%)**	**No**	**OR (95% CI)**	**I² (%)**
**Brain tumor**	**All**	23	0.99 (0.88 to 1.12)	59.8	5	1.32 (0.99 to 1.76)	67.9	6	0.80 (0.71 to 0.90)	32.6	12	1.02 (0.90 to 1.15)	0.0
**Malignancy**	**Malignant**	14	1.12 (0.95 to 1.32)	58.0	5	**1.35 (1.06 to 1.73)**	53.9	1	0.81 (0.70 to 0.94)	-	8	1.02 (0.86 to 1.21)	0.0
	**Benign**	15	0.87 (0.78 to 0.96)	17.3	3	0.94 (0.77 to 1.15)	0.0	5	0.79 (0.67 to 0.95)	46.0	7	0.97 (0.80 to 1.17)	0.0
**Type**	**Glioma**	12	1.07 (0.90 to 1.29)	54.1	3	1.40 (0.86 to 2.28)	76.1	1	0.81 (0.70 to 0.94)	-	8	1.04 (0.87 to 1.24)	0.0
	**Time since first use or latency ≥10 years**	3	1.66 (0.80 to 3.44)	81.0	1	3.60 (1.63 to 7.95)	-	1	0.98 (0.76 to 1.26)	-	1	1.61 (0.84 to 3.07)	-
	**Meningioma**	6	0.81 (0.72 to 0.90)	0.0	2	0.82 (0.64 to 1.06)	0.0	1	0.79 (0.68 to 0.91)	-	3	0.85 (0.64 to 1.12)	0.0
	**Acoustic neuroma**	6	1.00 (0.83 to 1.19)	13.9	2	1.20 (0.79 to 1.83)	0.0	1	0.85 (0.69 to 1.04)	-	3	1.10 (0.84 to 1.46)	6.7
	**Pituitary tumor**	3	0.71 (0.42 to 1.20).	71.5		n.a.		3	0.71 (0.42 to 1.20).	71.5		n.a.	
**Others**	**All**	14	0.98 (0.89 to 1.09)	0.0	5	1.03 (0.88 to 1.21)	0.0	3	0.85 (0.69 to 1.05)	0.0	6	1.03 (0.83 to 1.28)	37.5
**Malignancy**	**Malignant**	12	1.01 (0.91 to 1.13)	0.0	4	1.04 (0.88 to 1.22)	0.0	2	0.84 (0.54 to 1.31)	0.0	6	1.03 (0.83 to 1.28)	37.5
	**Benign**	3	0.88 (0.69 to 1.12)	0.0	1	2.51 (0.40 to 15.67)	-	2	0.86 (0.67 to 1.10)	0.0		n.a.	
**Type**	**Salivary Gland Tumor**	7	0.92 (0.78 to 1.09)	0.0	2	0.98 (0.70 to 1.39)	0.0	3	0.85 (0.69 to 1.05)	0.0	2	1.16 (0.74 to 1.82)	0.0
	**Leukemia & Lymphoma**	4	1.09 (0.92 to 1.28)	0.0	1	1.04 (0.79 to 1.37)	-		n.a.		3	1.12 (0.90 to 1.39)	14.2

**Melanoma**	2	0.84 (.60 to 1.20)	55.8	1	1.00 (0.73 to 1.30)	-		n.a.		1	0.70 (0.50 to 1.00)	-
**Testicular Cancer**	1	1.1 (0.8 to 1.5)	-	1	1.1 (0.8 to 1.5)	-		n.a.			n.a.	

n.a., not available; '*' indicates that cellular phone use statistically significantly increases the risk of tumor.
